# Catalytic Reductive Fractionation of Castor Shells into Catechols via Tandem Metal Triflate and Pd/C Catalysis

**DOI:** 10.3390/molecules31010120

**Published:** 2025-12-29

**Authors:** Jianan Hu, Weimin Zheng, Hao Li, Fuzhong Jiang, Jinlan Cheng, Bo Jiang, Tingwei Zhang, Chaofeng Zhang

**Affiliations:** 1 Jiangsu Co-Innovation Center of Efficient Processing and Utilization of Forest Resources, College of Light Industry and Food Engineering, Nanjing Forestry University, Nanjing 210037, China; 1998015@njfu.edu.cn (J.H.); jfznanlin@njfu.edu.cn (F.J.); nfucjl@njfu.edu.cn (J.C.); bjiang@njfu.edu.cn (B.J.); ztwei@njfu.edu.cn (T.Z.); 2 School of Pharmacy, Nanjing Medical University, 101 Longmi Avenue, Nanjing 211166, China

**Keywords:** lignin valorization, catalytic reductive fractionation, catechyl lignin, metal triflate catalysis, ether bond cleavage

## Abstract

In this work, the one-pot catalytic reductive fractionation of C-lignin in castor shell powders to efficiently provide catechyl monomers was achieved by tandem metal triflate and Pd/C catalysis. The optimized Pd/C + In(OTf)_3_ combination performed best and provided a 66.9 mg·g^−1^ yield of corresponding aromatic monomers with the catechol selectivity as high as 95.4%. For the promotion effect of the Lewis acid species, the mechanism studied indicated that the introduction of In^3+^ could significantly promote the C–O bond cleavage in the LCC to release the C-lignin fragments from the solid lignocellulose and simultaneously accelerate the cleavage of the critical C_α/β_–OAr linkage bond in C-lignin to release catechol monomers. In addition, performance differences highlight the cooperation and function-matching effect between the hydrogenation metals and the Lewis ion species, which can promote the high-value utilization of forestry and agricultural residues in chemical synthesis.

## 1. Introduction

In the current era, carbon cycling issues have become a hot topic of concern to the community, which calls for the development of sustainable biomass resource utilization to replace or supplement traditional fossil resources in the supply of chemicals and energy. Therefore, the transformation of renewable lignocellulose, especially for the non-edible lignocellulose or the corresponding residues from forestry and agricultural activities [1,2,3], to prepare chemicals has become increasingly important. The lignin, as one of the three main components of lignocellulose, consists of various phenylpropanoid units, which can be regarded as the unique aromatic bank for the preparation of functionalized aromatic chemicals directly [3,4,5,6,7,8]. The key issue for the lignin transformation/depolymerization lies in developing strategies with highly selective and active catalysts to effectively cleave the ubiquitous C–O/C–C bonds, leaving the aromatic benzene rings unconverted [9,10,11,12,13,14]. Although great efforts for lignin depolymerization to aromatic chemicals have been made in recent decades by efficiently cleaving the stable C–O/C–C linkage bonds, it remains a challenge for converting the isolated lignin efficiently and selectively, not to mention the direct conversion of the protolignin in the solid lignocellulose feedstock.

**Scheme 1:**
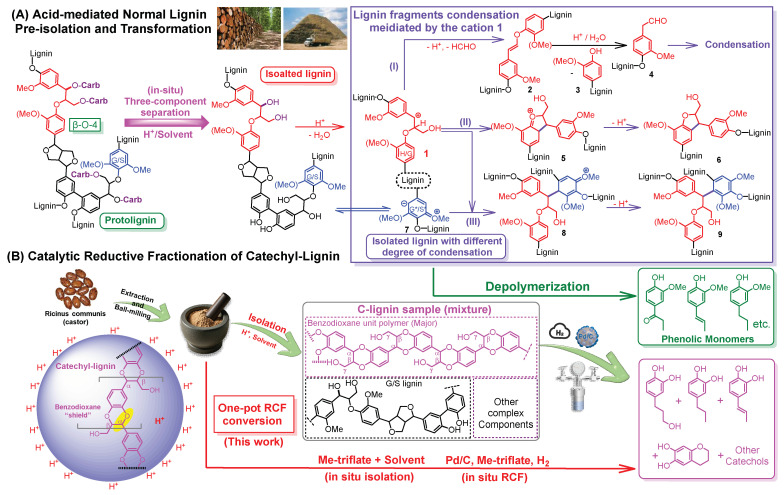
Acid-mediated normal G/S/H Lignin pre-isoaltion and transformation (**A**) and catalytic reductive fractionation of the acid-resistant catechyl lignin (**B**) via the multiple isolation-conversion process and the one-pot CRF of castor shells into catechol monomers over the Pd/C and metal triflate (this work).

So far, apart from direct use of industrial lignin derived from pulping for paper making, and lignocellulose enzymolysis for ethanol production, most of the current lignin conversion research has been carried out using pre-isolated lignin [3]. For selective lignin removal from intact lignocellulosic biomass, the most common method is organosolv processes, generally defined as pretreatments that employ an organic solvent, most commonly with an acid co-catalyst to liberate part of lignin from lignocellulosic biomass. Besides the issue of lignin extraction efficiency, given the typical requirement for acidity to cleave the weak C–O ether and ester bonds among lignin molecules and other components in lignocellulose to release the lignin fragments, the protonation of the C_α_–OH in various lignin structures can lead to the ready formation of reactive benzylic carbocations (Scheme 1A), which can readily participate in electrophilic aromatic substitution reactions on the electron-rich aryl groups of lignin to form recalcitrant C–C bonds [15,16]. In addition, the carbonyl compounds generated from acid-catalyzed lignin depolymerization can also undergo C–C bond condensation. As a result, non-native lignin-derived polymers are formed, which are less likely to be depolymerized. Furthermore, the isolated lignin fragments (supramolecular structure) without the solvent dispersion protection will undergo aggregation under the action of intermolecular forces such as hydrogen bonds, van der Waals forces, and electrostatic forces, which will increase the difficulty of lignin redispersion and depolymerization [17,18]. Therefore, the “lignin-first processing” [6], or in situ releasing-conversion of the native lignin fragments from the solid lignocellulose substrate, should be more efficient than the indirect conversion of isolated lignin. Even so, the “lignin-first” process of in situ lignin conversion still faces the problem of lignin structure repolymerization, because some acid catalysts are used to promote the release of lignin, as well as acidic species present in the catalysts or reaction systems. Therefore, for the efficient ‘lignin-first’ process, an extract stabilization approach needs to be carried out to prevent condensation reactions through either catalysis or protection-group chemistry. The emergence of the above-mentioned problems is mainly attributed to the high reactivity of the normal G or G/S type lignin structure (Scheme 1A). A potential solution is to use natural lignocellulose raw substrate containing pre-protected lignin structures.

Besides the normal G- or G/S-type lignin plants, some natural plants, such as vanilla, various members of the cactaceae, and castor, can contain an unusual catechyl-lignin (C-lignin) (Scheme 1B) due to their genetic characteristics [19,20,21,22,23]. The unique C-lignin was found to be essentially a homopolymer synthesized almost purely by β-O-4 coupling of caffeyl alcohol with the growing polymer chain, producing benzodioxanes as the dominant unit in the polymer (Scheme 1B). Because of the lack of an accessible and eliminable benzylic hydroxyl group in the C-lignin benzodioxane unit, condensation reactions due to the formation of benzyl cations might be mitigated under acidic conditions [22], which indicates the stability of C-lignin and the potential of C-lignin-containing biomass as the ideal substrate for the “lignin-first” transformation [22]. Besides the advantages of the chemical structure of C-lignin, the supply of the C-lignin-containing resource can also be guaranteed. The castor beans, as an important industrial resource for high-quality bio-oil production, achieved a total yield of 1.86 × 10^6^ tons in 2021, with India, Mozambique, Brazil, and China leading in production [24]. In addition, the endocarp of the castor shells, a by-product/waste from the castor processing industry, based on compositional analysis, is estimated to yield about 2.50 × 10^5^ tons of C-lignin, which is a plentiful and inexpensive feedstock for the production of more valuable catechol derivants used in bioactive molecules, drugs, and biomimetic functional materials [25,26,27,28,29,30,31]. Therefore, the full use of the C-lignin resource has drawn much attention [28,32,33,34].

By now, the C-lignin depolymerization to catechol monomers prefers the catalytic reductive fractionation (CRF) [32,35,36,37,38], mediated by the active metals, such as Pd, Pt, Ru, and Ni. In 2018, Li et al. achieved catechol-type monomers in near-quantitative yield with a selectivity of 90% to a single monomer via the Pd/C-mediated C-lignin hydrogenolysis at 200 °C and 4.0 MPa H_2_ [22]. Stone et al. investigated the depolymerization of C-lignin of vanilla seeds with a flow-through reductive catalytic fractionation system mediated by the Ni/C catalyst [39]. In 2021, Wang et al. reported that an atomically dispersed Ru/ZnO-C catalyst could efficiently achieve the hydrogenolysis of C-lignin to catechols with a unique selectivity to propenylcatechol (77%) [40]. Because most C-lignin conversion studies used pre-isolated C-lignin as the substrate, besides the conversion procedure, the isolation procedure could affect the following depolymerization. Besides the normal organic solvents, such as 1,4-dioxane, acetone, and alcohol, more efficient or selective systems, including the deep eutectic solvent (DES) [41,42,43], 2-MeTHF/H_2_O [44], maleic acid aqueous [45], and molten salt hydrate [46] were developed to disassemble catechyl lignin from castor shells. However, the direct conversion of C-lignin in the castor shells remains a challenging task [39]. In addition to the catalytic system, which could efficiently cleave the benzodioxane linkages, including the stable C_α/β_–OAr bonds [32], the in situ release of the C-lignin fragments from the compact castor shell structure was more challenging than from other wood or grass powders, during which the C-lignin, G/S lignin, cellulose, and hemicellulose are intricately cross-linked to constitute the castor shell lignocellulose.

In this work, focusing on the direct “lignin-first” transformation of the C-lignin from the solid castor shells, referring to the facts that the Lewis acid can effectively cooperate with alcohol solvent molecules to effectively eliminate the intermolecular forces and promote cleavage of weak C–O ether and ester bonds among multiple components of lignocellulose to release lignin fragments [47], and activate the stable C_α/β_–OAr bonds in lignin [48,49,50,51], the one-pot catalytic reductive fractionation of C-lignin in castor shell powders to efficiently provide catechyl monomers was studied by employing the tandem catalysis of Lewis metal salts and Pd/C. The optimized Pd/C + In(OTf)_3_ combination performed best and provided a 66.9 mg·g^−1^ yield of corresponding catechol monomers with the catechol selectivity as high as 95.4%. In addition, the promotion effects of the Lewis acid species, especially for the In^3+^, on the Pd/C-mediated C_α/β_–OAr bonds reductive cleavage in C-lignin and C-lignin in situ release were studied. In addition, performance differences highlight the cooperation and function-matching effect between the hydrogenation metals and the Lewis ion species. The current study, as complementary to previous reports, can offer a step-efficient protocol for producing valuable catechol compounds from bulk sustainable castor shells.

## 2. Results and Discussion

### 2.1. Composition of Castor Shells

**Figure 1:**
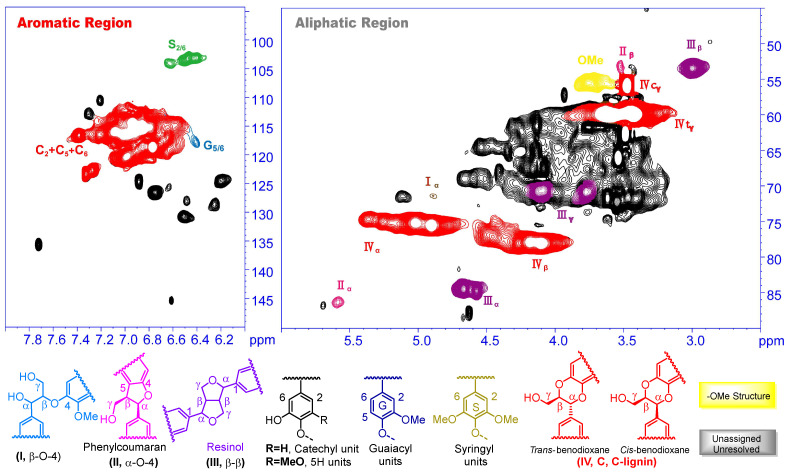
The aromatic region (**Left**) and aliphatic side-chain region (**Right**) of 2D NMR spectra of the C-lignin sample isolated from castor shell powders, and the typical lignin structure and units.

Given the unique stability of the benzodioxane structure in the C-lignin because of the lack of an accessible and eliminable benzylic hydroxyl group for the formation of benzyl cations, the catechyl lignin can survive and retain its original structure even with the harshest acidic pretreatment of the Klason (KL) isolation method [22]. We calculated the total lignin amount in the purchased castor shells using the Klason method after the benzene-ethanol extraction [42], which indicated that the extracted castor shell contains 61.7 wt% lignin with 60.1 wt% acid-insoluble Klason lignin and 1.6 wt% acid-soluble lignin, 19.7 wt% carbohydrate content (including glucose 10.7 wt% and xylose 9.0 wt%), and 3.8 wt% ash (Table S1). It can be seen from the data that the above lignin content of castor shells is significantly higher than that of other lignocellulosic substrates, and is overestimated than its actual lignin content, which may be related to the interference of other components like the residual proteins and fatty acids of the castor shells, the co-existence of G/S lignin, and other complex structure issues (Figure 1, black usassigned zones), or the compact lignocellulose structure in the castor shell [39,43,52]. Besides the Klason method, the thioacidolysis treatments were also used to release corresponding α,β,γ-trithioethylpropyl-substituted monomers from G/S lignin and C-lignin, providing the lignin content. While the benzodioxane unit of C-lignin is substantially resistant to thioacidolysis treatment (Figure S1), the C-lignin content was <5% checked by the thioacidolysis method in this work, which indicated that the real C-lignin content would be underestimated under such a measurement [23]. In addition, referring to previous reports [42,52], even for the isolated C-lignin samples obtained from different treatments, which can not guarantee the complete extraction of lignin, the purity of the isolated C-lignin is around 45~50 wt% based on the quantitative ^13^C NMR tests. Therefore, the content of C-lignin in castor shells is still rather difficult to determine accurately. In the following CRF tests, to improve the accuracy of quantitative analysis, this study adopted the quantitative unit of mg·g^−1^ to evaluate the generation of amomatic monomers from per gram of castor shells powder.

### 2.2. Catalytic Reductive Fractionation of Castor Shells to Aromatics over the Pd/C + Lewis Acid Systems

As discussed in the introduction, although C-lignin is extremely acid-resistant, even for the 72% sulfuric acid, the Lewis acid can effectively cooperate with alcohol solvent molecules to release lignin fragments from the solid lignocelluloses [47,53,54], and the metal ion with the appropriate ratio between the charge and ionic radius could coordinate with the nucleophilic oxygen atom and further induce the activation and cleavage of the C–O bond [48], the one-pot catalytic reductive fractionation (CRF) of C-lignin in castor shell powders to efficiently provide catechyl monomers was carried out.

**Figure 2:**
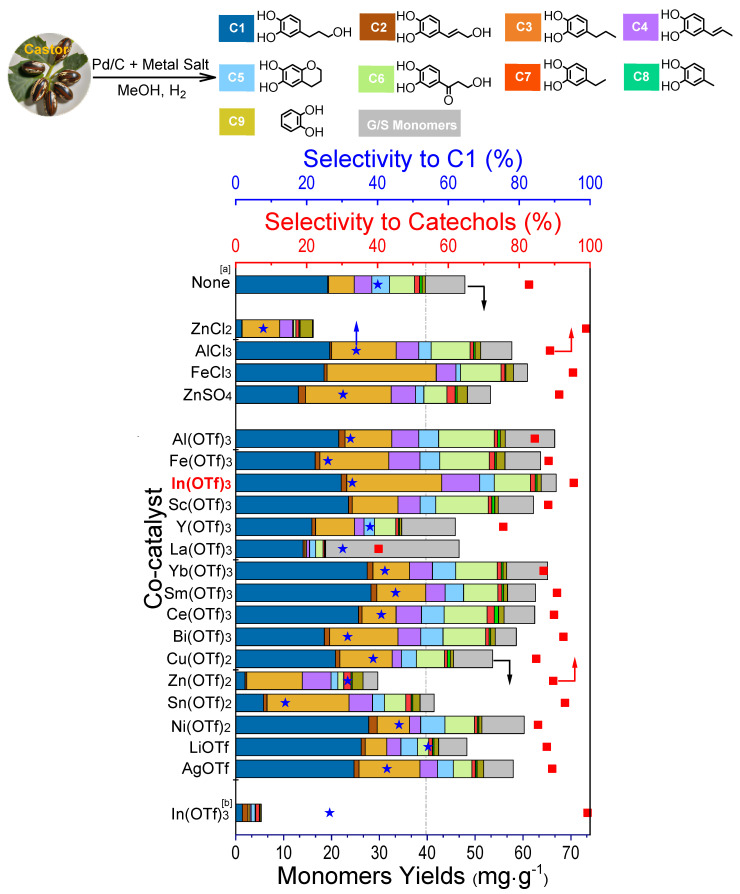
Influence of different Lewis salts on the Pd/C-mediated catalytic reductive fractionation of castor shells into catechyl monomers. Reaction conditions: Pre-extracted castor shell powders 100.0 mg, Pd/C 10.0 mg, co-catalyst (Lewis acid, metal cation) 0.01 mmol, MeOH 5.0 mL, H_2_ 2.5 MPa, 200 °C, 6 h. [a] Just Pd/C, no Lewis acid; [b] without Pd/C, just the In(OTf)_3_.

**Catalysts Screening.** The CRF of the castor shell powders mediated by the Pd/C was first carried out at 200 °C under the 2.5 MPa H_2_, which could provide a 47.8 mg·g^−1^ yield of aromatic monomers that induced 39.5 mg·g^−1^ of catechols (82.7% selectivity) and 40.1% selectivity towards the main product **C1** (4-(3-hydroxypropyl)benzene-1,2-diol) in 6 h (Figure 2). Further product analysis showed that the main monomers were generated from the cleavage of C_α/β_–OAr bonds in the C-lignin benzodioxane and the subsequent minor side chain breakage, and no obvious aromatic ring hydrogenation products were generated. Then, referring to the previous report that the Pd/C + ZnCl_2_ system could efficiently catalyze the celavgae of G/S lignin β-O-4 linkage for their unique cooperation mechanism [55], the catalytic performance of this combination was tested, which just provided a 16.2 mg·g^−1^ yield of aromatic monomers but with a 98.9% selectivity towards the catechols. Besides the divalent Zn^2+^ ion, the catalytic performance of trivalent metal cations of Al^3+^ and Fe^3+^ in their chloride form was tested. As shown in Figure 2, the AlCl_3_ provided a 57.7 mg·g^−1^ yield of aromatic monomers that induced 51.2 mg·g^−1^ of catechols (88.7% selectivity) and 34.0% selectivity towards the main product **C1**, and FeCl_3_ could provide a 60.9 mg·g^−1^ yield of aromatic monomers that induced 58.0 mg·g^−1^ of catechols (95.2% selectivity) and 30.3% selectivity towards the main product **C1** under the same condition. Compared with the single Pd/C performance, the above results confirmed the promotion effect of the Lewis acid in the C-lignin conversion, and the Lewis species with higher electrophilicity (or higher valence state to metal ion radius ratio) seem to have better catalytic activity. In addition, focusing on the reverse inhibition effect of C-lignin depolymerization catalyzed by the combination of ZnCl_2_ and Pd/C, which was the opposite of their promotion effect for the G/S lignin depolymerization [47], the influence of anions on the catalytic effect of Lewis ions was checked. When the ZnCl_2_ was replaced by the ZnSO_4_, the Pd/C + ZnSO_4_ system has a certain promoting effect compared with the Pd/C catalytic system, with the yield of aromatic monomers increased by 15.5% to 55.2 mg·g^−1^, but the selectivity of catechol monomers decreased to 91.3% with the obvious transformation of G/S lignin fragments. The reactivity differences caused by the above-mentioned ion/anion changes further demonstrated the influence of the Lewis acid species state on the catalytic depolymerization of C-lignin.

Furthermore, considering the stability issue of the common Lewis (chloride) under the relatively harsh reaction conditions (methanol and 200 °C) of this experiment, to further systematically verify the relevant conclusions about the ionic state influences of metal ion Lewis species on the Pd/C-mediated CRF of C-lignin with C–O linkage bonds cleavage, and based on the relatively stable and water-resistant properties of metal salts of metal triflate, this work further studied the catalytic reductive fractionation of castor shells into catechols mediated by the tandem catalysis of metal triflate and Pd/C (Figure 2). Compared with the catalytic performances of AlCl_3_, FeCl_3_, and ZnCl_2_, the monomers all increased when the corresponding metal salt anion was replaced with trifluoromethyl sulfonate. The Al(OTf)_3_ provided a 66.3 mg·g^−1^ yield of aromatic monomers with a 55.9 mg·g^−1^ yield of catechols, the Fe(OTf)_3_ provided a 63.7 mg·g^−1^ yield of aromatic monomers with a 56.2 mg·g^−1^ yield of catechols, and Zn(OTf)_2_ provided a 29.7 mg·g^−1^ yield of aromatic monomers with a 26.6 mg·g^−1^ yield of catechols. While the Zn(OTf)_3_ + Pd/C combination still had a lower monomer yield than that of the single Pd/C system (47.8 mg·g^−1^). In any case, the above results demonstrated that improving the stability of Lewis acids has an enhancing effect on the catalytic activity of the combined systems.

Then, the promotional effects of other metal triflates, with different valence states, were evaluated. Besides the metal ions, Zn^2+^, Sn^2+^, La^3+^, and Y^3+^, with significant inhibitory effects for the Pd/C-mediated castor shells CRF transformation, the catechol yield of other Pd/C + metal triflate systems rose with the valence state of the metal, which could be related to the enhancement of Lewis acidity with the increase in the charge of the transition metal cation. Among the various metal triflates, the In(OTf)_3_ + Pd/C provided the highest yields of aromatic monomers (66.9 mg·g^−1^) and catechols (63.8 mg·g^−1^), which increased by 40% and 61% compared with those of Pd/C alone, respectively. Meanwhile, the introduction of In(OTf)_3_ made the combined system more inclined to the transformation of C-lignin components, and the selectivity of catechol monomers reached 95.4%. In addition, although the acidity of In^3+^ ion alone was insufficient to effectively activate the critical C_α/β_–OAr linkage bonds of C-lignin, the 5.1 mg·g^−1^ yield of catechols confirmed that the benzodioxane structure was not unbreakable under the catalysis of the Lewis acid of In^3+^.

The above results further confirmed the important role and advantages of metal triflates in assisting Pd/C to efficiently catalyze the “lignin-first” CRF transformation of castor shells to catechols and regulate the product distribution. Meanwhile, the differences in reactivity and product distribution among the different Lewis species + Pd/C systems indicated that there was a corresponding function-matching mechanism between the hydrogenation metal centers (M^0^) and the corresponding Lewis acid species of metal ions (M’^n+^), especially for the combined In(OTf)_3_ + Pd/C system, which could affect the hydrogenation activity of the corresponding catalytic system and the transformation of critical interemdiate that further dominiated the monomers yield and production distribution (*vide infra*, C_α/β_–OAr bonds reductive cleavage and C-lignin in situ release).

**Figure 3:**
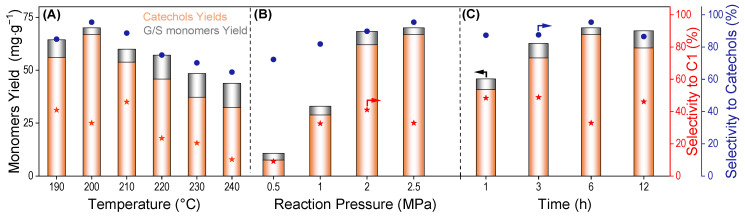
Influence of other reaction parameters. Reaction conditions: Pre-extracted castor shell powder 100.0 mg, Pd/C 10.0 mg, MeOH 5.0 mL, In(OTf)_3_ 0.01 mmol. (**A**) H_2_ 2.5 MPa, 3 h; (**B**) 200 °C, 6 h; (**C**) H_2_ 2.5 MPa, 200 °C.

**Investigation of Parameters.** Besides the synergistic effect of the Lewis acid + Pd/C RFC system, reaction temperature, H_2_ pressure, and reaction time can affect the reaction rate and dominate the final product yield and distribution, which are critical parameters for the lignin catalytic hydrogenolysis process and need to be optimized. Firstly, a suitable temperature can ensure the in situ release of lignin fragments from the solid lignocellulose, increase the kinetic energy of the reactant molecules, increase the frequency and energy of collision, and ultimately accelerate the reaction rate. As shown in Figure 3A, the yield of aromatic monomers first increased as the temperature rose. The In(OTf)_3_ + Pd/C system could provide a 55.9 mg·g^−1^ yield of catechol monomers with 40.9% selectivity to **C1** at 190 °C in 3 h, and the corresponding catechol monomer yield could increase by 19.6% to 66.9 mg·g^−1^ from 190 °C to 200 °C, with catechol selectivity increasing from 84.6% to 95.4%. Further increasing the reaction temperature to 210 °C caused a decrease in the catechol monomers yield to 53.8 mg·g^−1^, but accelerated the depolymerization of G/S lignin, which resulted in a decrease in the catechols selectivity to 88.6%. Further increasing the temperature above 210 °C could further induce both decreases in the monomer yield and catechol monomers selectivity, even though 210 °C provided the highest **C1** selectivity at 46% (Figure 3A).

In addition, the addition of H_2_ provided as the hydrogen source for the hydrogenolysis reaction was necessary, because using methanol as the hydrogen source alone was not sufficient to support the CRF reaction mediated by the Pd/C and In(OTf)_3_ at 200 °C, which provided a <0.2 mg·g^−1^ yield of catechol monomers (Scheme S1). When the H_2_ pressure gradually increased, the catechol monomers yield increased significantly in the range of 0–2 MPa (Figure 3B), which provided a 55.7 mg·g^−1^ (89.8 mol% catechols selectivity and 41.1 mol% **C1**) yield of catechols at 2.0 MPa H_2_. Further increasing the H_2_ pressure to 2.5 MPa, the catechol monomers yield slightly increased to 63.8 mg·g^−1^ (95.4% catechols selectivity), but the main catechol monomer (**C1**) selectivity decreased to 32.9%, compared with the value of 2.0 MPa. Furthermore, appropriate reaction time can control the final product yield and distribution (Figure 3C). The reaction at 1.0 h with a 45.9 mg·g^−1^ yield of phenolic monomers and an 87.3% selectivity towards catechol monomers. As the reaction time increased, the yield of catechol monomers from 1 h to 3 h increased by 36.9% to 55.7 mg·g^−1^, and the yield of catechol monomers from 3 h to 6 h further increased by 20% to 66.9 mg·g^−1^. Then, the catechol monomer yield slightly decreased to 60.5 mg·g^−1^ when the reaction time reached 12 h. In conclusion, further increasing the temperature, raising the H_2_ pressure or prolongs the reaction time will lead to a decline in the yield or selectivity of catechol, especially the selectivity of the main product **C1**, which may be attributed to the uncontrollability of bond breaking and the generation-transformation of critical intermediates under long-term high-temperature and harsh conditions, resulting in the accelerated occurrence of side reactions. Therefore, the optimal reaction conditions for the CRF transformation of castor shells into catechyl monomers mediated by tandem catalysis In(OTf)_3_ and Pd/C were determined to be 2.5 MPa H_2_, 200 °C, and 6 h, which provided the highest yield of aromatic monomers and the catechol monomer selectivity.

### 2.3. The Mechanism of In^3+^ on the Pd/C-Mediated C_α/β_–OAr Bonds Reductive Cleavage and C-Lignin In Situ Release

As discussed above, the catechyl monomers were the main aromatic product from the hydrogenative depolymerization of castor shell powders, which involved the selective and efficient cleavage of the C_α_–OAr and C_β_–OAr bond in the benzodioxane unit of C-lignin. To further provide an understanding of the promotion effect of the Lewis acid In^3+^ on the Pd/C-mediated hydrogenative cleavage of the C_α/β_–OAr bonds in C-lignin, the catalytic conversion of the C-lignin model compounds, including benzyl phenyl ether (BPE, α-O-4 model), 2-phenethyl phenyl ether (2-PPE, β-O-4 model), and (1-phenylethane-1,2-diyl)bis(oxy))dibenzene (PBOD, α-O-4 + β-O-4), over the In(OTf)_3_, Pd/C, and Pd/C + In(OTf)_3_ systems were checked.

As shown in Table 1, entries 1 and 4, the In(OTf)_3_ alone provided an unobvious C_α/β_–OAr bond cleavage in BPE and 2-PPE at the setting temperatures, while the Pd/C could provide a 51.5% conversion of BPE at 35 °C in 10 min (entry 2), and provide a 63.1% conversion of the 2-PPE at 180 °C for 360 min (entry 5). Furthermore, the combination of the Pd/C + In(OTf)_3_ could provide an 81.5% conversion of BPE at 35 °C in 10 min (entry 3), but a similar conversion of 2-PPE (89.8%) was needed to increase the reaction temperature to 180 °C and react for 360 min (entry 6). Therefore, besides the fact that the hydrogenative cleavage of the C_α_–OAr bond in BPE was easier than the C_β_–OAr bond in 2-PPE, based on the reaction temperature and time, there was an obvious synergistic effect between the Pd/C and Lewis acid In^3+^ in the hydrogenative cleavage of the C_α/β_–OAr ether bonds. In addition, when the C_α/β_–OAr bonds in the same molecule, such as the substrate PBOD, the Pd/C could primarily cleave the C_α_–OAr bond and provide an 54.1% conversion of PBOD with a 49.3% yield of 2-PPE, a 46.8% yield of phenol, and just 3.1% yield of methylbenzene at 90 °C for 60 min (entry 8). Furthermore, the Pd/C + In(OTf)_3_ system provided an 81.6% conversion of PBOD, with the main product of 2-PPE and phenol after the primary cleavage of the C_α_–OAr bond (entry 9). The above results further confirmed the cleavage order of the ether linkage bonds in the benzodioxane unit and indicated a synergistic effect between the Pd/C and Lewis acid In^3+^ in the hydrogenative cleavage of the C_α/β_–OAr ether bonds. Based on the BDE of the corresponding C_α_–OAr bond (56.5 kcal/mol) and C_β_–OAr bond (63.4 kcal/mol), it could be reasonable that the benzodioxane unit hydrogenolysis begins with the first cleavgae of the C_α_–OAr bond to C_α_H_2_, followed by the hydrogenolysis of the C_β_–OAr bond to C_β_H_2_ with the generation of catechol monomers, during which the Lewis In^3+^ species played a critical role in the activation of the C–O bond [48].

**Figure 4:**
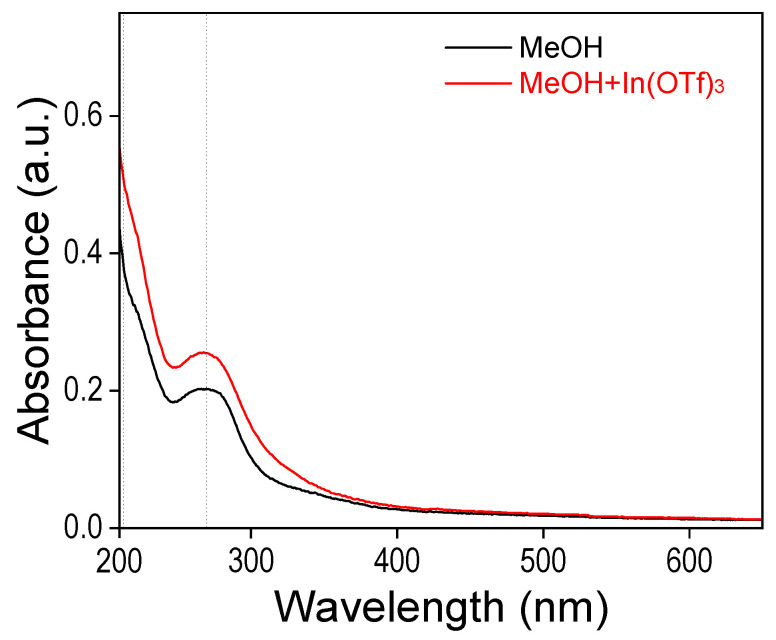
Influence of Lewis acid In(OTf)_3_ addition on the C-lignin in situ releasing from the solid castor shell. Reaction conditions: Pre-extracted castor shell powders 100.0 mg, In(OTf)_3_ 0.01 mmol, MeOH 5.0 mL, Ar 1.0 MPa, 6 h, 200 °C.

In addition, given the fact that lignocellulose samples are complexes formed by the interaction of different high-molecular structures through chemical bonds, intermolecular forces, van der Waals forces, electrostatic interactions, hydrogen bonds, etc. [3]. Therefore, the arrayed aliphatic OH groups on linear C-lignin in the castor shell may bind with other biopolymers, such as cellulose, hemicellulose, proteins, etc., not including the normal G/S lignin, based on the previous report [23]. As a result, the real heterogeneous conversion of protolignin first involves the dissolution of C-lignin molecular fragments from solid lignocellulose raw materials [42,56]. Based on the previous report [47], the Lewis acid could provide a better performance in the in situ release of the lignin fragment from the solid lignocellulose with the assistance of the organic solvent. The influence of Lewis acid In(OTf)_3_ addition on the C-lignin in situ releasing from the solid castor shell was carried out by checking the lignin concentration with the UV–Vis spectrometer. As shown in Figure 4, when the castor shell powders were treated with hot MeOH at 200 °C under an Ar atmosphere, there was a typical UV–Vis absorption peak at 270 nm [57], which can be attributed to the aromatic rings of the C-lignin fragments in situ dissolved by the methanol solvent and the potential furan structure generated from the carbohydrates of the lignocellulose. Based on the determining experience of acid-dissolved lignin by the Klason method, the relatively accurate content of soluble lignin could be determined based on the light absorption at 205 nm, where the light absorption of the furan ring is relatively weak. Furthermore, when the Lewis acid In(OTf)_3_ was added to the extraction system, the concentration of the in situ released C-lignin from the solid castor shell increased 27.7% based on the absorbance at 205 nm. Therefore, besides the promotion effect of the Lewis ion in the C-lignin C_α/β_–OAr ether bond activation, the Lewis acid species could accelerate the C-lignin in situ release from the solid castor shell poders. [47] As a coin has two sides, the Lewis metal ion addition can also cause the slight degradation of cellulose and hemicellulose to furan-based oligomers.

**Figure 5:**
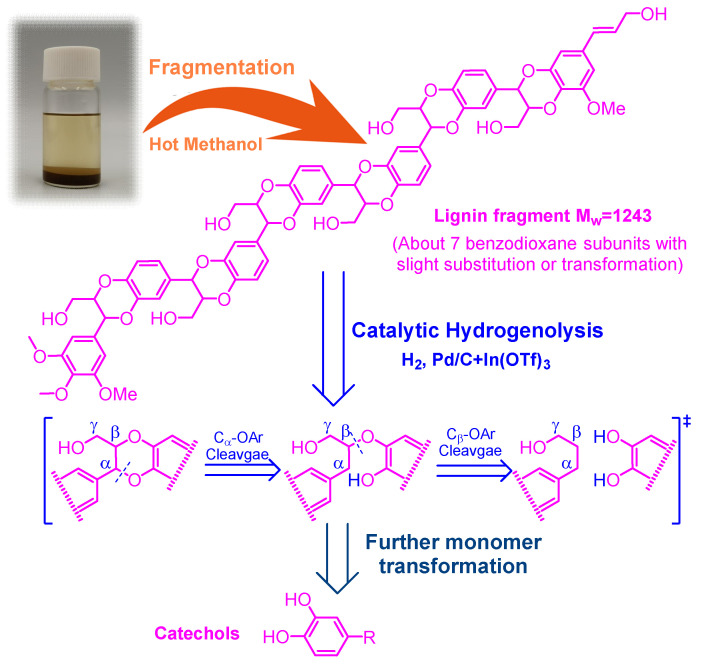
The in situ releasing of the C-lignin fragments from the solid castor shells and its in situ CRF transformation into catechyl monomers mediated by the Lewis acid In(OTf)_3_ and Pd/C.

As shown above, the hot methanol solvent dominated the in situ lignin fragments content released from the solid castor shell powders. For the characterization of the released C-lignin fragments, MODIL-TOF analysis was performed. As shown in Figure S1, the solid castor shell first provided the oligomer with eight catechol phenolic units in the hot methanol, according to the MS peaks and the structure prediction. According to Song’s report [56], for the heterogeneous catalytic conversion of solid lignocellulose, the soluble oligomers released under the assistance of solvents are more likely to come into contact with the heterogeneous catalytic centers and are the critical species mediating the CRF process. Therefore, it was the C-lignin oligomer generated from the in situ isolation from solid castor shell powders that mediated the further hydrogenative depolymerization to catechols (Figure 5). Furthermore, during the following fragmentation of the C-lignin oligomer, the synergistic effect between the Lewis acid In^3+^ and Pd/C centers played critical roles in the selective and efficient cleavage of the C_α_/_β_–OAr ether bonds of the benzodioxane linkage, and the released monomers could further undergo hydroxyl hydrogenolysis, hydroxyl dehydration-isomerization, and cyclization to generate the corresponding catechol monomers [40] listed in Figure 2.

### 2.4. Catalytic Reductive Fractionation of Castor Shells to Aromatics over the Ni/C + Lewis Acid Systems

The above results demonstrated the advantages of specific Lewis acid species combined with Pd/C in the “lignin-first” transformation of castor powders into catechols. To further verify the function-matching issue and synergistic effect, between specific Lewis acid species and metal hydrogenation centers, and taking into account the cost issue brought about by the use of noble metal catalysts in the real lignin conversion and utilization process of castor powder, this study initially carried out the exploration of “lignin-first” conversion of castor powders with Lewis acid species-assisted non-precious metal hydrogenation catalysts.

**Figure 6:**
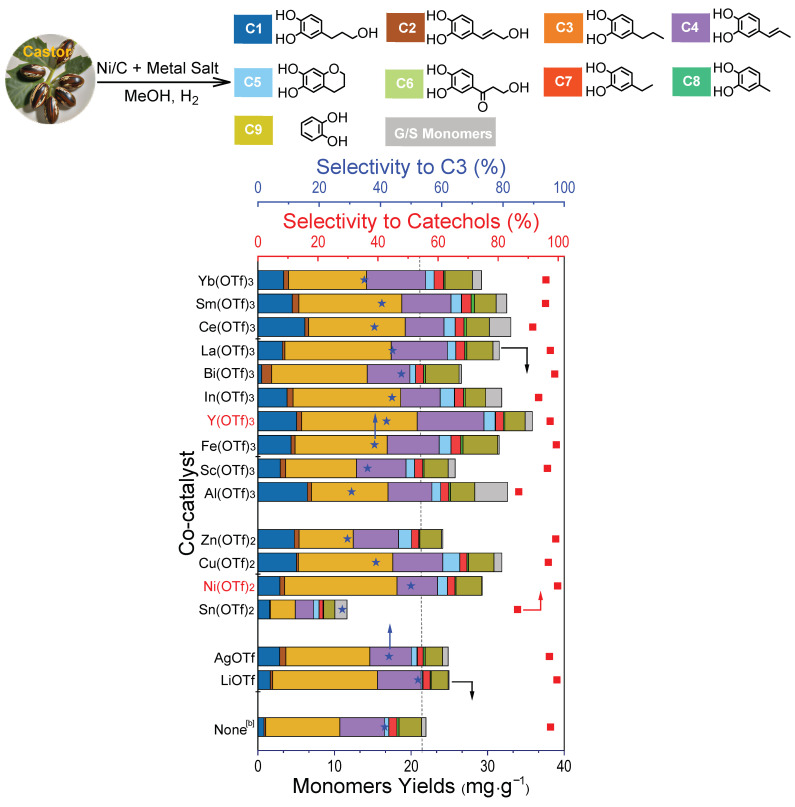
Influence of different Lewis salts on the Ni/C-mediated catalytic reductive fractionation of castor shells into catechyl monomers. Reaction conditions: Pre-extracted castor shell powder 100.0 mg, Ni/C (5 wt%) 12.5 mg, co-catalyst 0.01 mmol, MeOH 5.0 mL, H_2_ 2.5 MPa, 200 °C, 6 h. [a] Just Ni/C.

Among the numerous non-precious metal heterogeneous catalysts, Ni/C catalysts have received extensive attention in the research of lignin or direct lignocellulose CRF conversion due to their efficient and low-cost preparation methods and unique reactivity regulation properties [58,59]. The Ni/C catalyst prepared by the carbothermic reduction was selected to conduct the “lignin-first” transformation of castor powders (Figure 6). As shown in Figure 6, the Ni/C alone just provided a 23.4 mg·g^−1^ yield of aromatic monomers, including 22.8 mg·g^−1^ yield of catechols, whose overall monomer yield was lower than Pd/C (47.8 mg·g^−1^). While the Ni/C-catalyzed CRF transformation and catechyl monomers generated from the Ni/C-catalyzed castor shells CRF transformation were more inclined to undergo hydrogenolysis of the side chain (-C_γ_H_2_OH) hydroxyl group, among which the monomer selectivity of C_3_ (4-propylbenzene-1,2-diol) could reach 41.4%. The overall product yield and the product distribution differences could be attributed to the different catalytic hydrogenation capabilities and reaction selectivity of Ni/C and Pd/C. Furthermore, when different metal Lewis acid species were introduced into the Ni/C-catalyzed CRF system, unlike Pd/C + M^n+^ systems, which exhibited different catalytic effects associated with the addition of metals, the introduction of most metal triflates, except for the Sn(OTf)_2_, could increase the yields of aromatic monomers and catechol monomers to a certain extent. Among the Ni/C + M^n+^ systems, Ni/C + Y(OTf)_3_ provided the highest yields of aromatic monomers (53.4% increase to 35.9 mg·g^−1^) and catechol monomers (53.5% increase to 35.0 mg·g^−1^) compared with Ni/C. Meanwhile, when correlating the transformation results with different metal valences, it was found that the higher metal valence state was more conducive to increasing the catechol yield for the Ni/C + metal triflate systems. Furthermore, although the catalytic hydrogenation ability of Ni/C was weaker than that of Pd/C, resulting in a lower monomer yield, the yield of catechol monomers in the Ni/C system was significantly higher. Meanwhile, under the regulatory effect of appropriate Lewis acid species, such as Ni(OTf)_2_, Li(OTf), and Fe(OTf)_3_, the Ni/C + metal triflate systems can achieve the priority conversion of C-Lignin to catechols from the C-Lignin and G/S lignin mixed substrate. The Ni/C + Ni(OTf)_2_ system just provided a 29.4 mg·g^−1^ yield of phenolic monomers from the CRF transformation of castor shells, but the selectivity towards catechol products reached 99.8%, and the selectivity of C3 was 50 mol%. The similar promotion and regulation effects could be found in the Ni/C + LiOTf system, which provided a high selectivity towards catechols (99.6%) but a lower monomer yield (26.2 mg·g^−1^). In addition, the Ni/C + Fe(OTf)_3_ could provide a higher monomer yield (31.6 mg·g^−1^) with the selectivity towards catechols at 99.4%.

Based on the above CRF experimental results catalyzed by Ni/C and Pd/C, adding appropriate metal Lewis species to the hydrogenation systems could not only enhance the transformation efficiency by strengthening the corresponding synergistic effect, but also provide new means for the preferential transformation of lignin substrates and the regulation of reaction products, which were critical issues for lignin valorization.

## 3. Materials and Methods

### 3.1. Materials, Chemicals, and Catalysts

The chemicals, including various metal salts, Pd catalyst, benzene, ethanol, methanol, dodecane, N, O-bis (trimethylsilyl) trifluoroacetamide (BSTFA), and model compounds including benzyl phenyl ether (BPE), 2-phenethyl phenyl ether (2-PPE), and (1-phenylethane-1,2-diyl)bis(oxy)dibenzene (PBOD), were purchased from commercial suppliers in analytical grade without further purification. The castor seeds were purchased from the local pharmacy. The castor shells were first peeled and crushed to 40–60 mesh, and then packaged and placed into a Soxhlet extractor. The castor shell powders were then extracted with benzene and ethanol (1:2) at 90 °C for 8 h to remove the lipids, waxes, fats, etc., after which the dried solid was further extracted with hot water for 5 h. The obtained castor shell powders, after the ethanol washing and vacuum drying, were further subjected to 9 h of ball milling in a planetary ball mill. The obtained castor sample was then kept sealed for further use.

The Pd content of the commercial catalyst Pd/C was 10 wt%. The Ni/C was prepared via the carbothermic reduction method [60]. In detail, the commercial activated carbon (5.0 g) was added to the Ni(NO_3_)_2_ solution (8.0 mL), and the beaker was covered with a Petri dish to keep the sample moist for 24 h. Subsequently, the samples were dried overnight at 120 °C. Then, the solid was reduced in a horizontal furnace in a N_2_ flow (30 mL·min^−1^) at 450 °C for 2 h. The metal loading of the Ni/C catalyst was 5 wt%.

### 3.2. Procedure for Catalytic Hydrogenolysis

In a typical reaction, the pre-extracted castor shell powders (100.0 mg), catalysts including Pd/C (10.0 mg), metal salt (0.01 mmol), and solvent (methanol, 5.0 mL) were added to the high-pressure reactor with magnetic stirring (25.0 mL). The reactor, after sealing, was charged with 1.0 MPa N_2_ three times to replace the air and then filled with H_2_ to the specified pressure. Then, the reactor was heated to the specified temperature in a heating module. After the reaction, the reactor was cooled down with the assistance of the machine fan before releasing the gas. After the primary filtration treatment, the residue was washed with 10.0 mL of methanol, and the organic liquids were combined to obtain the lignin oil by removing the remaining solvent with the rotary evaporator. Then, an external standard (dodecane) in the anhydrous THF (5.0 mL) was added to the lignin oil, which was then treated with extra BSTFA (0.30 mL) at 65 °C for 1 h under N_2_. The final products after the second filtration treatment were analyzed by the GC-MS (GC: Shimadzu 2030AM, MS: Shimadzu, QP2020NX). The monomer yield and selectivity were calculated with the following formulas:

(1) $$\mathrm{Monomer}\;\mathrm{yield}\;(\mathrm{mg}\cdot g^{-1})\;=\;\frac{\mathrm{Catechol}\;\mathrm{monomers}\;\mathrm{yields}\;(\mathrm{mg})}{\mathrm{Castor}\;\mathrm{shell}\;\mathrm{powders}\;(g)}$$

(2) $$\mathrm{Selectivity}\;\mathrm{to}\;\mathrm{catechol}\;\mathrm{monomers}\;(\%)=\frac{\mathrm{Catechol}\;\mathrm{monomers}\;\mathrm{yields}\;(\mathrm{mg})}{\mathrm{Total}\;\mathrm{monomers}\;\mathrm{yield}\;(\mathrm{mg})}\times 100\%\;$$

Note: In the above equations, the monomer yields were based on the yield of catechol monomers (mg) per gram of extracted castor shell powder sample, which contains benzodioxane polymers (C-lignin), normal G/S lignin, and other carbohydrate structures.

## 4. Conclusions

In conclusion, the one-pot catalytic reductive fractionation of C-lignin in castor shell powders to efficiently provide aromatic monomers, mainly composed of catechols, was achieved by tandem metal triflate and Pd/C catalysis. Experimental results showed that the introduction of Lewis acids could significantly increase the yield of catechol monomers in the Pd/C-mediated hydrogenolysis system by assisting the cleavage of the critical C_α/β_–OAr linkage bond in C-lignin to release catechol monomers and simultaneously promote the C–O bonds cleavage in the LCC to accelerate C-lignin release from the solid lignocellulose substrate. Among the Lewis metal ions, the catalytic performance of trivalent metals was superior to that of divalent metals, and that of divalent metals was better than that of monovalent metals. In addition, compared with the conventional metal chlorides that have a certain promoting effect on the yield increase, metal trifluoromethanesulfonates have a more significant promotion on the yield of catechol monomer, and the combination of the Pd/C + In(OTf)_3_ performed best with a 66.9 mg·g^−1^ yield of corresponding catechol monomers and the catechol selectivity as high as 95.4%. Besides the Pd/C, the Lewis acid-promoting strategy was also used in the Ni/C-mediated CRF of castor shell powders. Although the yields of the Ni/C system were lower than the Pd/C systems, the optimized Ni/C + Ni(OTf)_2_ system could provide a 99.8% selectivity towards the catechol monomers, and the monomer yield reached 29.4 mg·g^−1^. The above results highlight the cooperation and function-matching adjustment effects between the hydrogenation metal catalysts and the Lewis ion species, which can promote the high-value utilization of forestry and agricultural residues in chemical synthesis.

## Figures and Tables

**Table 1:** Catalytic hydrogenolysis of BPE, 2-PPE, and BPOD over the Pd system with the In(OTf)_3_.

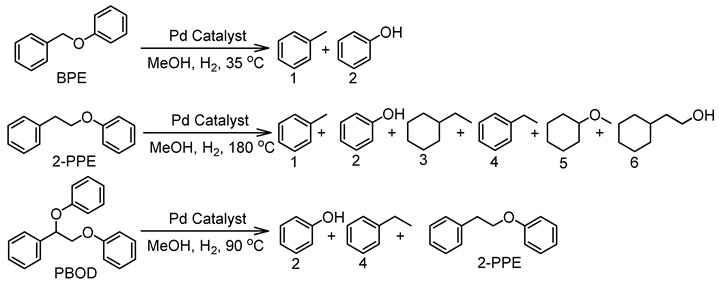										
Entry	Substrate	Catalyst	Conversion (%)	Products Distribution (%)						
				1	2	3	4	5	6	2-PPE
1	BPE ^a^	In(OTf)_3_	-	-	-	-	-	-	-	-
2	BPE ^a^	Pd/C	51.5	24.0	26.3	-	-	-	-	-
3	BPE ^a^	Pd/C + In(OTf)_3_	81.5	35.5	46.0	-	-	-	-	-
4	2-PPE ^b^	In(OTf)_3_	-	-	-	-	-	-	-	-
5	2-PPE ^b^	Pd/C	63.1	11.9	3.0	3.0	15.2	25.6	4.4	-
6	2-PPE ^b^	Pd/C + In(OTf)_3_	89.8	15.7	12.1	4.3	20.5	31.5	5.6	-
7	PBOD ^c^	In(OTf)_3_	-	-	-	-	-	-	-	-
8	PBOD ^c^	Pd/C	54.1	-	46.8	-	3.1	-	-	49.3
9	PBOD ^c^	Pd/C + In(OTf)_3_	81.6	-	74.4	-	4.1	-	-	71.3

Reaction conditions: Substrate 0.2 mmol, Pd/C(10 wt%) 10.0 mg, In(OTf)_3_ 0.01 mmol, methanol 5.0 mL, H_2_ 0.2 MPa. ^a^ 35 °C, 10 min; ^b^ 180 °C, 360 min; ^c^ 90 °C, 60 min.

## Data Availability

Data are contained within this article or the Supplementary Materials.

## Supplementary Materials

The following supporting information can be downloaded at: https://www.mdpi.com/article/10.3390/molecules31010120/s1, Table S1: Compositional analysis of castor shells, Figure S1: Products GC-MS spectrum of castor shells thioacidolysis, Figure S2: The MODIL-TOF spectrum of the in-situ released C-lignin from the castor shell powders, Figure S3: ^1^H-NMR and ^13^C-NMR of PBOD, Scheme S1: The CRF transformation of castor shells without H_2_.
