# Supplementary material for: Catalytic Reductive Fractionation of Castor Shells into Catechols via Tandem Metal Triflate and Pd/C Catalysis
Source: Molecules. 2025 Dec 29;31(1):120. doi: 10.3390/molecules31010120 (PMC12787612; doi:10.3390/molecules31010120)
Supplement: Supplementary file 1 [file molecules-31-00120-s001.zip › molecules-4011261-supplementary.pdf]

molecules

MDPI

Supporting Information

Catalytic Reductive Fractionation of Castor Shells into Catechols via Tandem Metal Triflate and Pd/C Catalysis

Jianan Hu,[a] Weimin Zheng,[b] Hao Li,[a] Fuzhong Jiang,[a] Jinlan Cheng,[a] Bo Jiang,[a] Tingwei Zhang,[a] and Chaofeng Zhang*[a]

[a] J.A. Hu, H. Li, F.Z. Jiang, Dr. B. Jiang, Dr. J. Cheng, Prof. Dr. C.F. Zhang,

Jiangsu Co-Innovation Center of Efficient Processing and Utilization of Forest Resources, College of Light Industry and Food Engineering, Nanjing Forestry University, Nanjing 210037 (P.R. China)

[b] Dr. W.M. Zheng

School of Pharmacy, Nanjing Medical University, 101 Longmi Avenue, Nanjing 211166, China

Corresponding Authors:

Prof./Dr. Chaofeng Zhang, Email: zhangchaofeng@njfu.edu.cn.

Page number 8, Figure number 3, Table number 1

Table of Contents

1. Characterizations ······ S3
2. Solubility of C-lignin in Methanol Solution ······ S3
3. Compositional Analysis of Castor Shells ······ S3
4 Castor Shells Powders Thioacidolysis ······ S4
5 The CRF Transformation of Castor Shells without H_2 ······ S5
6 The MODIL-TOF Spectrum of the in situ Released C-lignin ······ S5
7 NMR Spectra of the Model Compound PBOD ······ S6
8 References ······ S7

https://doi.org/10.3390/molecules31010120

1. Characterizations

The 2D HSQC NMR spectra of isolated lignin and lignin oil were characterized by an AVANCEIIIHD 600MHz nuclear magnetic resonance (NMR) spectrometer by dissolving the isolated C-lignin sample (50.0 mg) in dimethyl sulfoxide (DMSO)-d_6 (0.50 mL). The MALDI-TOF spectra of the C-lignin sample were recorded by the Bruker UltrafleXtreme TOF/TOF.

2. Solubility of C-lignin in Methanol Solution

The castor powder (100 mg) was added to the hydrogenation reactor twice with 5 mL of methanol. During the first time, no $In(OTf)_3$ was added, and during the second time, 0.01 mmol of $In(OTf)_3$ was added. Then the mixture was treated at 200 °C for 6 h under 1.0 MPa Ar. After the treatment and cooling naturally, the liquid components were collected via centrifugation, and the solid was washed with methanol. The two-part liquid was added to a 25 mL volumetric flask, and extra methanol was added to the flask to the mark. Then, the UV-vis spectra (200-800 nm) of the two samples were obtained over a Shimadzu UV-2600 UV-vis spectrophotometer.

3. Compositional Analysis of Castor Shells

Table S1. Compositional analysis of castor shells

Composition	wt%
Lignin content	**61.7**
Klason lignin	60.1
Acid-soluble lignin	1.6
Carbohydrate content	**19.7**
Glucose	10.7
Xylose	9.0
Ash	**3.8**

https://doi.org/10.3390/molecules31010120

4. Castor Shells Powders Thioacidolysis

Thioacidolysis treatments can release the corresponding α, β, γ-trithioethylpropyl-substituted monomers from G/S lignin, which can be detected by GC/GC-MS.[1] However, the C-lignin content was <5% checked by the thioacidolysis method in this work, which seemed unreasonable. This could be attributed to the fact that the benzodioxane bond in C-lignin is highly resistant to thioacidolysis treatments. Therefore, the actual C-lignin content could be underestimated via such measurement.[2]

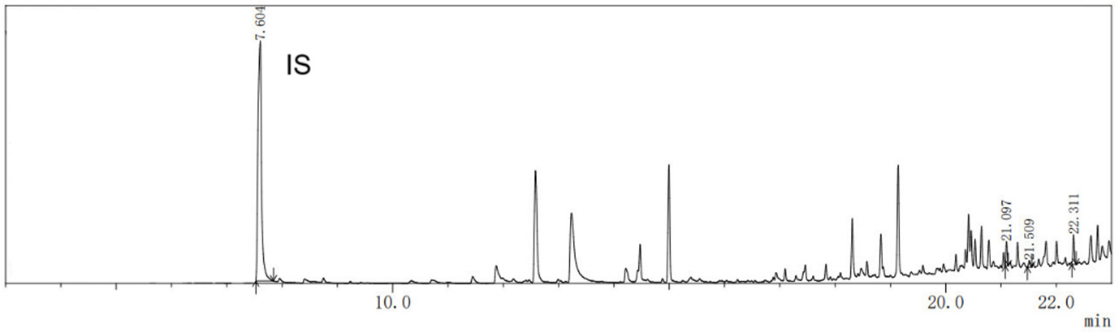

Figure S1. Products GC-MS spectrum of castor shells thioacidolysis

Note: Thioacidolysis analysis was performed according to the previous report.[1c,2] In brief, 30 mg of sample (seed coats powders) was treated with BF_3-etherate (0.2 M, 5 mL) in a dioxane/ethanethiol (8.75:1, v/v) at 100 °C for 4 h. The reaction mixture was extracted with dichloromethane. After the removal of all volatiles of the organic phase under vacuum, the resulting residue was dissolved in anhydrous THF containing an internal standard (dodecane), which was treated with N, Obis(trimethylsilyl)trifluoroacetamide (BSTFA) at 65 °C for 1 h under N_2 before GC-MS analysis.

https://doi.org/10.3390/molecules31010120

5. The CRF Transformation of Castor Shells without H_2

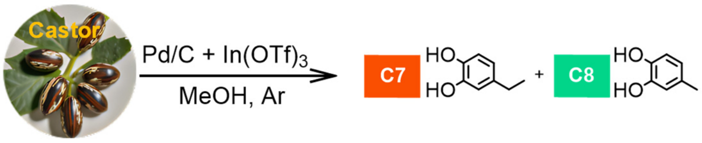

Yield: <0.2 mg·g^{-1}; **C7:** 0.07 mg·g^{-1}; **C8:** 0.01 mg·g^{-1}

Scheme S1. The CRF transformation of castor shells without H_2. Reaction condition: Castor shell powder 100.0 mg, Pd/C 10.0 mg, MeOH 5.0 mL, $In(OTf)_3$ 0.01 mmol, Ar 1 atm, 200 °C, 6 h.

6. The MODIL-TOF Spectrum of the in situ Released C-lignin

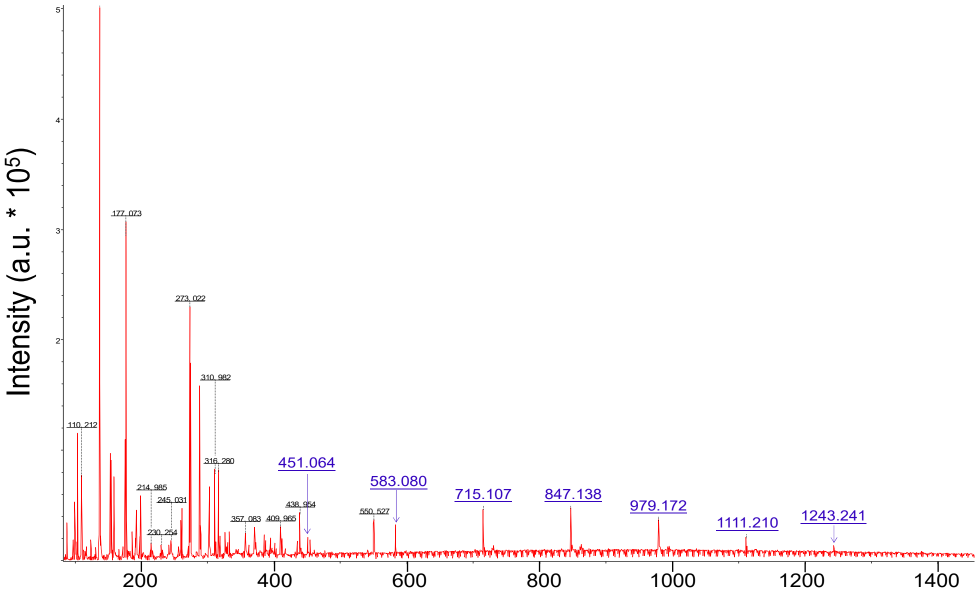

Figure S2. The MODIL-TOF spectrum of the in-situ released C-lignin from the castor shell powders. Reaction condition: Extracted castor shell powders 100 mg, MeOH 10.0 mL, Ar 1.0 MPa, 190 °C, 3 h.

According to the molecular weight of the basic benzodioxane unit in the C-lignin (M_w=164), the largest m/z=1243 peak in Figure S2 suggests that the corresponding C-lignin fragment should contain 7 benzodioxane units.

https://doi.org/10.3390/molecules31010120

7. NMR Spectra of the Model Compound PBOD

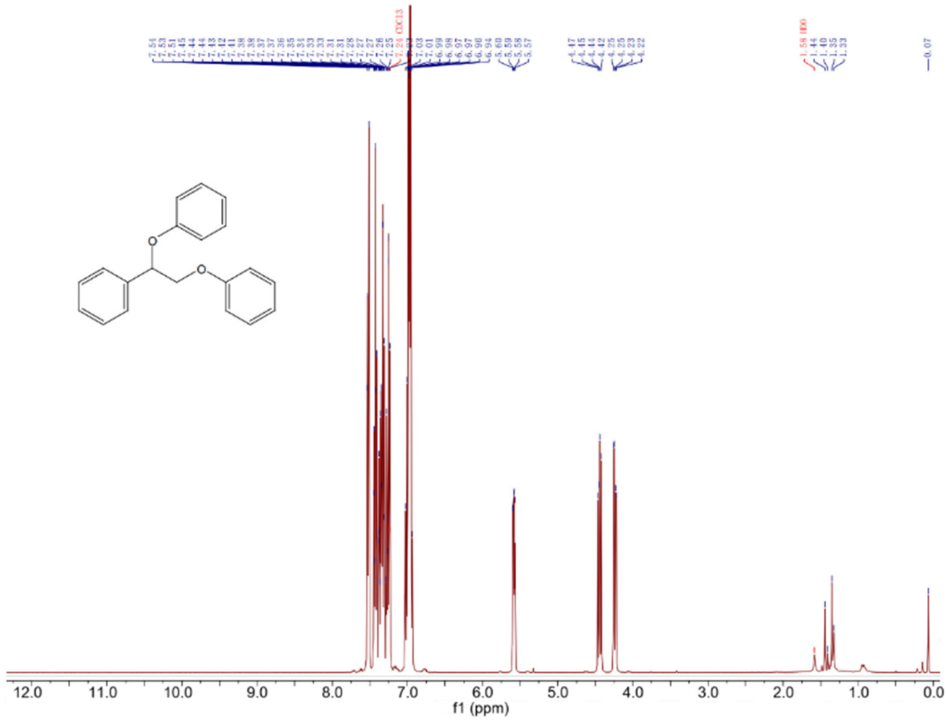

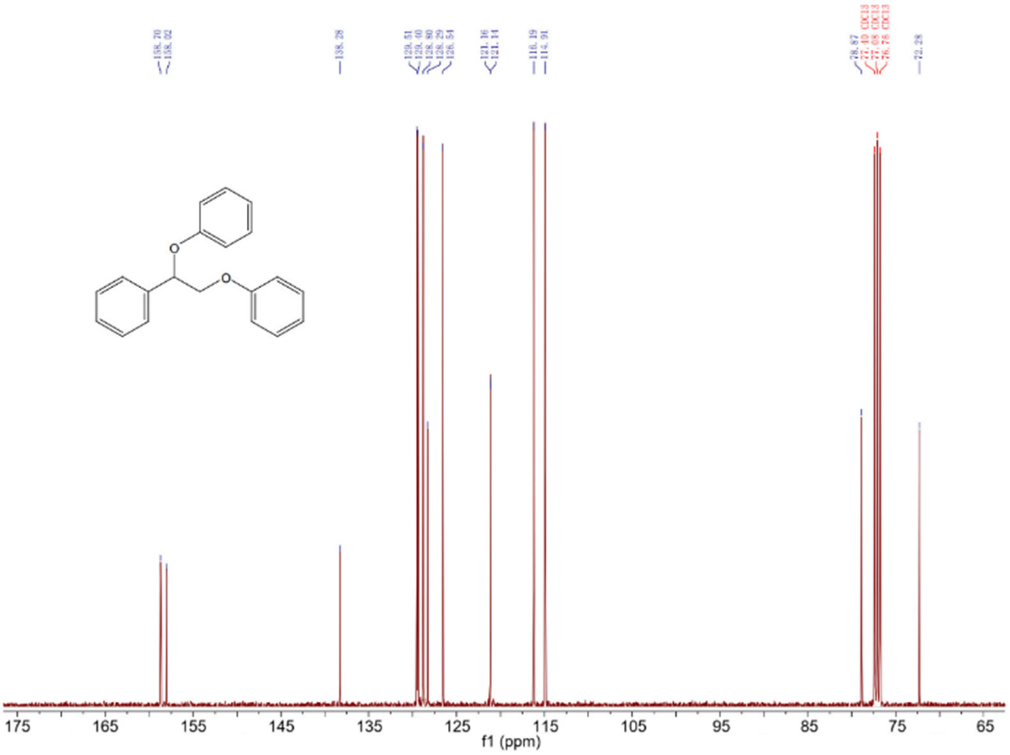

Figure S3. ^{1}H-NMR and ^{13}C-NMR of PBOD

NMR data: ^{1}H NMR (400 MHz, $CDCl_3$) δ 7.52 (d, *J* = 7.1 Hz, 2H), 7.47 – 7.21 (m, 7H), 7.05 – 6.91 (m, 6H), 5.58 (dd, *J* = 7.7, 3.8 Hz, 1H), 4.44 (dd, *J* = 10.3, 7.7 Hz, 1H), 4.24 (dd, *J* = 10.3, 3.8 Hz, 1H). ^{13}C NMR (100 MHz, $CDCl_3$) δ 158.70, 158.02, 138.28, 129.51, 129.40, 128.80, 128.29, 126.54, 121.16, 121.14, 116.19, 114.91, 78.87, 72.28.

https://doi.org/10.3390/molecules31010120

8. References

[1] (a) S. Z. Wang; S. H. Su; L. P. Xiao; B. Wang; R. C. Sun; G. Y. Song. *ACS Sustainable Chem. Eng.* **2020,** *8*, 7031-7038; (b) Y. Li; L. Shuai; H. Kim; A. H. Motagamwala; J. K. Mobley; F. Yue; Y. Tobimatsu; D. Havkin-Frenkel; F. Chen; R. A. Dixonet al. *Sci. Adv.* **2018,** *4*, eaau2968; (c) S. Su; S. Wang; G. Song. *Green Chem.* **2021,** *23*, 7235-7242.

[2] Y. Tobimatsu; F. Chen; J. Nakashima; L. L. Escamilla-Trevino; L. Jackson; R. A. Dixon; J. Ralph. *Plant Cell* **2013,** *25*, 2587-2600.
